# Studies on citrullinated LL-37: detection in human airways, antibacterial effects and biophysical properties

**DOI:** 10.1038/s41598-020-59071-7

**Published:** 2020-02-11

**Authors:** Salma Al-Adwani, Cecilia Wallin, Melanie D. Balhuizen, Edwin J. A. Veldhuizen, Maarten Coorens, Michael Landreh, Ákos Végvári, Margaretha E. Smith, Ingemar Qvarfordt, Anders Lindén, Astrid Gräslund, Birgitta Agerberth, Peter Bergman

**Affiliations:** 1 Department of Laboratory Medicine, Division of Clinical Microbiology, Karolinska Institutet, Stockholm, Sweden; 2 Department of Animal and Veterinary Sciences, College of Agricultural and Marine Sciences, Sultan Qaboos University, Muscat, Oman; 3 Department of Biochemistry and Biophysics, Stockholm University, Stockholm, Sweden; 4 Department of Infectious Diseases and Immunology, Division of Molecular Host Defence, Faculty of Veterinary Medicine, Utrecht University, Utrecht, The Netherlands; 5 Department of Microbiology, Tumor and Cell Biology, Karolinska Institutet, Stockholm, Sweden; 6 Division of Physiological Chemistry I, Department of Medical Biochemistry & Biophysics, Karolinska Institutet, Stockholm, Sweden; 7 Department of Internal Medicine and Clinical Nutrition, Institute of Medicine at Sahlgrenska Academy, University of Gothenburg, Gothenburg, Sweden; 8 Unit for Lung and Airway Research, Institute of Environmental Medicine, Karolinska Institutet, Stockholm, Sweden; 9 Department of Respiratory Medicine and Allergy, Karolinska University Hospital, Stockholm, Sweden; 10 Infectious Disease Clinic, Immunodeficiency Unit, Karolinska University Hospital, Stockholm, Sweden

**Keywords:** Biophysics, Immunology, Microbiology

## Abstract

Arginine residues of the antimicrobial peptide LL-37 can be citrullinated by peptidyl arginine deiminases, which reduce the positive charge of the peptide. Notably, citrullinated LL-37 has not yet been detected in human samples. In addition, functional and biophysical properties of citrullinated LL-37 are not fully explored. The aim of this study was to detect citrullinated LL-37 in human bronchoalveolar lavage (BAL) fluid and to determine antibacterial and biophysical properties of citrullinated LL-37. BAL fluid was obtained from healthy human volunteers after intra-bronchial exposure to lipopolysaccharide. Synthetic peptides were used for bacterial killing assays, transmission electron microscopy, isothermal titration calorimetry, mass-spectrometry and circular dichroism. Using targeted proteomics, we were able to detect both native and citrullinated LL-37 in BAL fluid. The citrullinated peptide did not kill *Escherichia coli* nor lysed human red blood cells. Both peptides had similar α-helical secondary structures but citrullinated LL-37 was more stable at higher temperatures, as shown by circular dichroism. In conclusion, citrullinated LL-37 is present in the human airways and citrullination impaired bacterial killing, indicating that a net positive charge is important for antibacterial and membrane lysing effects. It is possible that citrullination serves as a homeostatic regulator of AMP-function by alteration of key functions.

## Introduction

Antimicrobial peptides and proteins (AMPs) are essential components of innate immunity with broad-spectrum antimicrobial activity against bacteria, fungi and viruses^1^. AMPs are actively produced and secreted by epithelial cells and phagocytes and contribute to clearing mucosal surfaces from pathogenic microbes and elimination of intracellular pathogens^2^. In addition, AMPs are also known as host defence peptides (HPDs) since they exhibit immunomodulatory activities, such as acting as signaling molecules and with the capacity to recruit neutrophils and macrophages to sites of infection. HDPs display both pro- and anti-inflammatory activities depending on the context^3,4^.

The cathelicidin family of AMPs has been detected in all mammals currently studied. AMPs of the cathelicidin family are produced with a conserved N-terminal cathelin domain and a variable C-terminal domain which after proteolysis, constitutes the active antimicrobial peptide^5^. Until date, LL-37 is the only cathelicidin that has been identified in humans and it has antimicrobial activity against both Gram-positive and Gram-negative bacteria^6–8^. LL-37 is a cationic and amphipathic peptide, composed of 37 amino acids (Table 1), it binds to the negatively charged membranes of bacteria, leading to disruption of the bacterial membrane integrity, which in turn causes bacterial death^9,10^. In model membrane systems composed of large unilamellar phospholipid vesicles (LUVs), LL-37 was found to cause membrane leakage from negatively charged vesicles but not from zwitterionic vesicles^11^. Furthermore, LL-37 has immunomodulatory properties, such as neutralization of lipopolysaccharide (LPS)-induced macrophage activation, chemoattractant activity towards immune cells^12,13^ and activation of autophagy^14^.

**Table 1:** The sequences of the synthetic LL-37 peptides used in this study and their physiochemical characteristics.

Peptide	Sequence	Mw (Da)	Net charge
LL-37	LLGDFF**R**KSKEKIGKEFK**R**IVQ**R**IKDFL**R**NLVP**R**TES	4493.4	6+
LL-37_Cit1_	LLGDFF**(Cit)**KSKEKIGKEFK**R**IVQ**R**IKDFL**R**NLVP**R**TES	4494.25	5+
LL-37_Cit2_	LLGDFF**(Cit)**KSKEKIGKEFK**(Cit)**IVQ**R**IKDFL**R**NLVP**R**TES	4495.23	4+
LL-37_Cit3_	LLGDFF**(Cit)**KSKEKIGKEFK**R**IVQ**R**IKDFL**(Cit)**NLVP**(Cit)**TES	4496.4	3+
LL-37_Cit5_	LLGDFF**(Cit)**KSKEKIGKEFK**(Cit)**IVQ**(Cit)**IKDFL**(Cit)**NLVP**(Cit)**TES	4498.19	1+

Mw, molecular weight (average mass); **R**, arginine residue; (Cit), citrulline residue.

Notably, under inflammatory conditions, calcium-dependent peptidyl arginine deiminase enzymes (PADs) are expressed in the same location as the human cathelicidin LL-37^15^. These enzymes catalyse citrullination, a post-translational modification (PTM) in which positively-charged arginine residues are converted into citrulline, which reduces the charge of targeted proteins or peptides^12^. In humans, there are five isotypes of PADs (PAD1, 2, 3, 4 and 6), which differ in their tissue distribution^16^. PAD2 and PAD4 are both expressed in neutrophils, where PAD2 is located in the cytosol and PAD4 is primarily located in the nucleus^17^. PAD4 is involved in the formation of neutrophil extracellular traps (NETs) and NETosis by citrullinating the positively charged histone proteins, leading to chromatin decondensation^18^. Importantly, *in vitro* studies have demonstrated that recombinant PAD2 and PAD4 causes different degrees of citrullination for arginine residues in LL-37, which results in a reduced overall peptide charge and abrogation of its LPS neutralizing activity^15,19^.

Given the broad specificity of PAD enzymes and its co-expression during inflammation in the lungs, it is reasonable to suggest that LL-37 can serve as a substrate for PAD-activity in the lungs. However, citrullinated LL-37 has not yet been detected in samples from human lungs and the putative role of citrullinated versions of LL-37 remains poorly defined. We therefore hypothesized that citrullination may occur in human lungs and that this PTM alters the microbiological and biophysical functions of LL-37. Thus, we designed a series of experiments to test this hypothesis by applying targeted protein analysis to human bronchoalveolar lavage (BAL) fluid obtained from healthy human volunteers, after intra-bronchial exposure to LPS, with the aim to identify citrullinated versions of LL-37. In addition, we determined the antimicrobial and biophysical properties of the synthetic citrullinated peptide by bacterial killing assays, transmission electron microscopy (TEM), isothermal titration calorimetry (ITC), mass spectrometry (MS) and circular dichroism (CD) and compared these properties with the native LL-37 peptide.

## Results

### Citrullinated LL-37 is present in BAL fluid from healthy volunteers after intra-bronchial exposure to LPS

**Figure 1:**
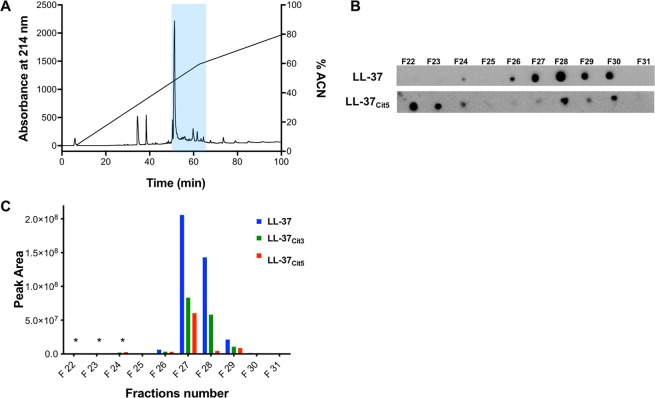
Identification of citrullinated LL-37 in BAL fluid from healthy volunteers exposed to LPS in the lungs. Healthy human volunteers were exposed to LPS and after 24 h, BAL fluid samples were collected. **(A)** Reversed-phase chromatography of 0.5 mg peptide/protein extract from the BAL fluid samples. Absorbance at 214 nm is indicated on the left Y-axis and concentration of acetonitrile (ACN) on the right Y-axis. The blue shaded area indicates fractions F22 to F31. **(B)** Dot blot detection of native and citrullinated LL-37 in the fractions of the BAL fluid extract using a monoclonal antibody against native LL-37 and a polyclonal serum raised against LL-37_Cit5_. **(C)** Fractions of the BAL fluid extract (F22-F31) were analyzed using the LC-ESI-MS-PRM method. Endogenous fragment ions unique for native LL-37, LL-37_Cit3_ and LL-37_Cit5_ were clearly detected in fractions (F22-F31) with different intensities of each peptide (Table 2). Asterisks (*) designate the detection of fragment ions corresponding to native LL-37, LL-37_Cit3_ and LL-37_Cit5_ in very low intensities (see Table S1).

Given that native LL-37 and PAD4 are highly expressed in the human lung during inflammation^15,20^, we set out to identify citrullinated variants of LL-37 under these conditions. We utilized cell-free BAL fluid samples obtained from healthy volunteers (n = 10) intra-bronchial exposure to LPS, which was pooled and fractionated using reversed-phase chromatography into 62 fractions (F1-F62) (Fig. 1A). To screen for variants of LL-37 peptides, dot blot analysis of the fractions was employed utilizing a monoclonal anti-LL-37 antibody or polyclonal serum raised against LL-37_Cit5_ (Fig. 1B). A signal corresponding to native LL-37 was detected in fractions F26 – F30. Notably, the screening also revealed a signal corresponding to LL-37_Cit5_ in fractions F22-F24 and F28-F30. To verify these findings and to rule out cross-reactivity, fractions F22 - F31 were further processed using a targeted LC-MS/MS approach. First, synthetic LL-37, LL-37_Cit3_ and LL-37_Cit5_ were analyzed by ESI-MS-PRM to investigate their ionization and fragmentation properties. Based on these observations, a set of 15 unique fragment ions with high charge states were selected to monitor longer fragment ions (Table 2). The set of fragment ions were used to identify native and different variants of citrullinated LL-37 peptides (Fig. 1C). The results showed a clear detection of native LL-37 in fractions F26-F29, whereas LL-37_Cit3_ and LL-37_Cit5_ were detected in the same fractions. Fragment ions corresponding to native LL-37, LL-37_Cit3_ and LL-37_Cit5_ were also found in fractions F22-F24, but with much lower intensities (asterisks in Fig. 1C, Table S1). Thus, based on the MS analysis, both native and citrullinated versions of LL-37 peptides were detected in cell-free BAL fluid obtained from healthy volunteers exposed to LPS.

**Table 2:** The detected 15 most intensive fragment ions of native and citrullinated LL-37 peptides analyzed by the LC-ESI-MS-PRM method.

Fragment ions	LL-37: 749.4365, 6+		LL-37 cit3: 899.7128, 5+		LL-37 cit5: 900.1064, 5+	
	*m/z*	Sequence	*m/z*	Sequence	*m/z*	Sequence
y7	801.4465+	LVPRTES				
78^*^	**915.4894**+	NLVPRTES	**915.4894**+	NLVPRTES	**916.4734**+	NLVP**R**TES
y9^*^	**1071.5905**+	RNLVPRTES	**1071.5905**+	RNLVPRTES		
y11^*^	**1331.7430**+	FLRNLVPRTES	**1331.7430**+	FLRNLVPRTES	**1333.7111**+	FL**R**NLVP**R**TES
y13	787.9361++	KDFLRNLVPRTES				
y14	844.4781++	IKDFLRNLVPRTES				
y15	922.5287++	RIKDFLRNLVPRTES				
y16	986.5580++	QRIKDFLRNLVPRTES				
y17			1036.5842++	VQ**R**IKDFLRNLVPRTES	1037.5682++	VQ**R**IKDFL**R**NLVP**R**TES
y18	1092.6342++	IVQRIKDFLRNLVPRTES				
y21	872.5134++++	FKRIVQRIKDFLRNLVPRTES				
y22	915.5276+++	EFKRIVQRIKDFLRNLVPRTES				
y23	958.2259+++++	KEFKRIVQRIKDFLRNLVPRTES			959.5380+++	KEFK**R**IVQ**R**IKDFL**R**NLVP**R**TES
y24	977.2331+++	GKEFKRIVQRIKDFLRNLVPRTES	977.8891++++	GKEFK**R**IVQ**R**IKDFLRNLVPRTES	978.5451+++	GKEFK**R**IVQ**R**IKDFL**R**NLVP**R**TES
y25			1015.5838+++	IGKEFK**R**IVQ**R**IKDFLRNLVPRTES		
y26					1058.9381+++	KIGKEFK**R**IVQ**R**IKDFL**R**NLVP**R**TES
b8^*^	**977.5567+**	LLGDFFRK	**978.5407+**	LLGDFF**R**K	**978.5407+**	LLGDFF**R**K
b9			1065.5728+	LLGDFF**R**KS	1065.5728+	LLGDFF**R**KS
b10			1193.6677+	LLGDFF**R**KSK	1193.6677+	LLGDFF**R**KSK
b11			1322.7103+	LLGDFF**R**KSKE	1322.7103+	LLGDFF**R**KSKE
b12			1450.8053+	LLGDFF**R**KSKEK	1450.8053+	LLGDFF**R**KSKEK
b19			1155.6521++	LLGDFF**R**KSKEKIGKEFK**R**	1155.6521++	LLGDFF**R**KSKEKIGKEFK**R**
b22	883.5181+++	LLGDFFRKSKEKIGKEFKRIVQ				
b23			936.5359+++	LLGDFF**R**KSKEKIGKEFK**R**IVQ**R**	936.5359+++	LLGDFF**R**KSKEKIGKEFK**R**IVQ**R**
b25			1016.9289+++	LLGDFF**R**KSKEKIGKEFK**R**IVQ**R**IK	1016.9289+++	LLGDFF**R**KSKEKIGKEFK**R**IVQ**R**IK
b26			1055.2712+++	LLGDFF**R**KSKEKIGKEFK**R**IVQ**R**IKD	1055.2712+++	LLGDFF**R**KSKEKIGKEFK**R**IVQ**R**IKD

y, C-terminal peptide fragment ions; b, N-terminal fragment ions; **R**, citrullinated arginine; *, reporter fragment detected in different forms.

### Citrullination abolishes the antimicrobial and membrane perturbing effects of LL-37

**Figure 2:**
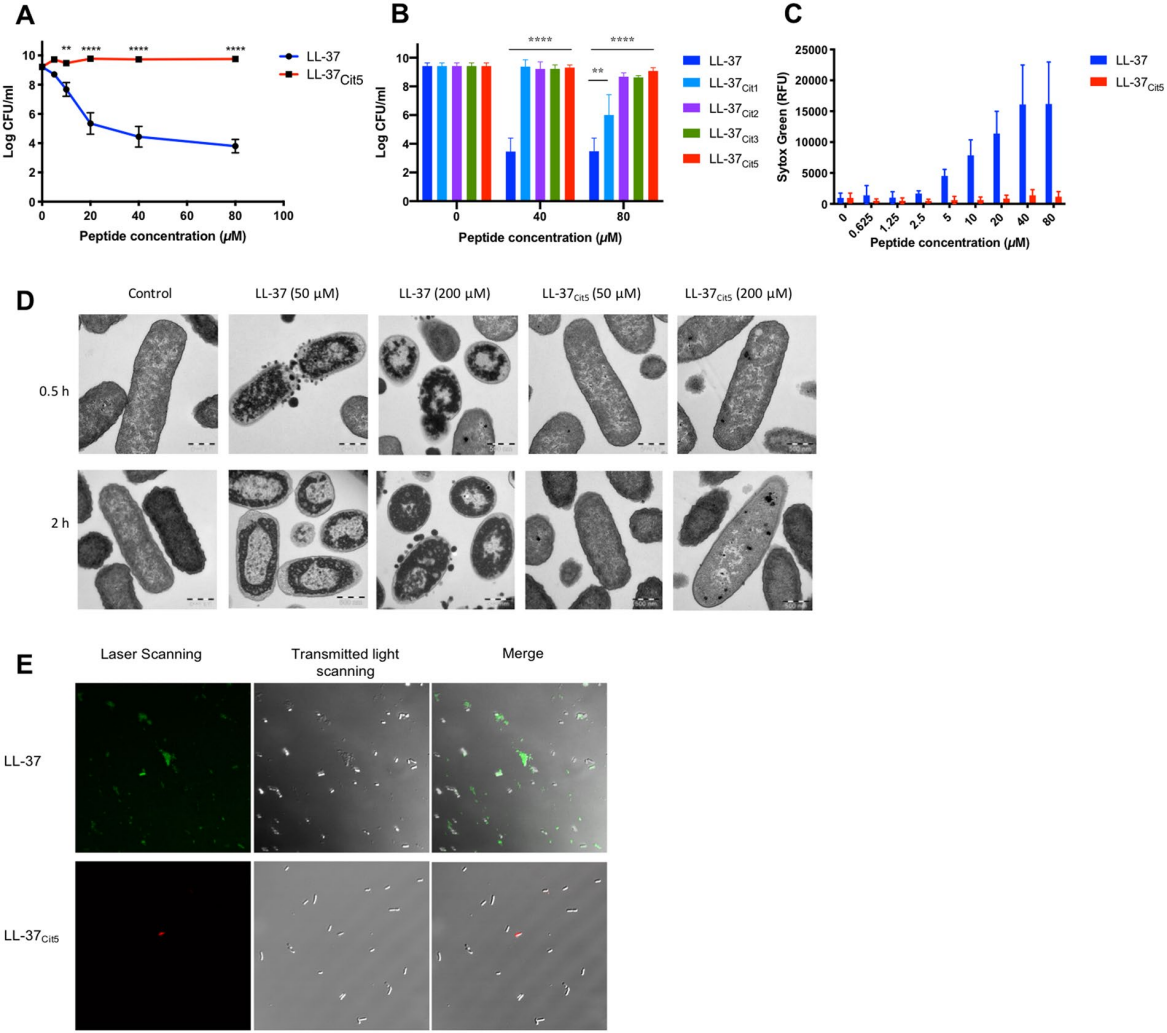
Antibacterial and membrane-disruptive properties of native and citrullinated LL-37. **(A)** *E. coli* ATCC 29522 (5 × 10^7^ CFU/ml) was co-incubated with different concentrations of native LL-37 and fully citrullinated LL-37 peptides or (**B**) partially citrullinated peptides at 37 °C for 3 hours after which bactericidal activity was determined by CFU (data representative of 3 independent experiments). **(C)** *E.coli* (5 × 10^7^ CFU/ml) was mixed with either native LL-37 or LL-37_Cit5_ (0–80 µM), followed by analysis of the inner membrane permeabilization by Sytox Green nucleic acid staining (data representative of 3 independent experiments). **(D)** Transmission electron microscopy images of 5×10^8^ CFU/mL of *E. coli* left untreated or treated with native LL-37 or LL-37_Cit5_ (50 and 200 µM) for 0.5 or 2 hours. Scale bars, 500 nm. **(E)** Confocal microscopy of 10^6^ CFU/mL of *E. coli* was mixed with fluorescently labeled LL-37 or LL-37_Cit5_ at 40 µM for 1 hour in LB (data representative of 2 independent experiments). Error bars show SEM. Statistical significance was evaluated by two-way ANOVA **(A**) or multiple t-test (**B**): *p-*values represented by stars; (<0.05*, <0.01**, <0.001***, <0.0001****).

Knowing that native LL-37 was present in human BAL fluid^21^, we determined the impact of citrullination on bacterial killing. As expected, synthetic native LL-37 (Table 1) exhibited potent antibacterial activity against *E. coli* and reduced colony count units (CFU) levels in a dose-dependent manner. In contrast, the fully citrullinated peptide LL-37_Cit5_ lacked antibacterial activity (Fig. 2A). Notably, citrullination of one arginine residue was enough to abolish the antibacterial activity at 40 μM, however at an increased concentration (80 μM) the peptide with one single citrullination reduced bacterial growth by 3 log units (Fig. 2B). In the following experiments in this study we compared the native peptide with the fully citrullinated peptide (LL-37_Cit5_) unless otherwise stated. Next, the capacity of the peptides to induce membrane leakage was tested using the permeability marker sytox green which detects nucleic acids. In *E. coli*, native LL-37 caused membrane permeability in a dose-dependent manner, starting from 2.5 μM up to 80 μM. In contrast, citrullinated LL-37 did not cause leakage at concentrations from 2.5–10 μM, whereas a slight permeability was observed at 20–80 μM concentrations (Fig. 2C). To visualize how native and citrullinated LL-37 peptides interacted with *E. coli*, transmission electron microscopy (TEM) studies were performed (Fig. 2D). Overall, non-treated bacteria had intact membranes and an even intracellular distribution of DNA and ribosomes in the cytoplasm (light and darker areas, respectively). After 30 min, exposure to 50 μM native LL-37 resulted in release of membrane vesicles and caused intracellular changes, such as clustering of DNA and ribosome condensations compared to non-treated bacteria. After 2 h treatment, a distinct segregation of DNA into the center of the cell was observed, whereas the ribosome clusters became denser and were directed towards the inner membrane. At a peptide concentration of 200 μM, native LL-37 compromised membrane integrity and increased ribosome clustering and cell lysis. In contrast, treatment with 50 μM LL-37_Cit5_ for 30 min and 2 h, did not result in any morphological changes. Only at a high concentration (200 μM) of LL-37_Cit5_, a few ribosomal clusters were observed in some bacteria, indicating cellular stress (Fig. 2D). To evaluate the ability of LL-37_Cit5_ to bind to the surface of intact bacteria, fluorescence-labelled peptides were incubated with *E. coli* for 1 hour. Rhodamine-B-LL-37_Cit5_ was unable to interact with *E. coli* in comparison to 5-FAM-LL-37 which also caused cell rupture and release of cell debris as seen in the merged panel of confocal microscopy images (Fig. 2E). To study the effects of citrullinated LL-37 on eukaryotic cell membranes, the hemolytic activity of citrullinated LL-37 was determined using human red blood cells. The native LL-37 hemolyzed approximately 8% of erythrocytes at 20 μM, whereas citrullinated LL-37 did not cause any hemolysis (Fig. S1).

### Native LL-37 and LL-37Cit5 exhibit different LPS binding properties

**Figure 3:**
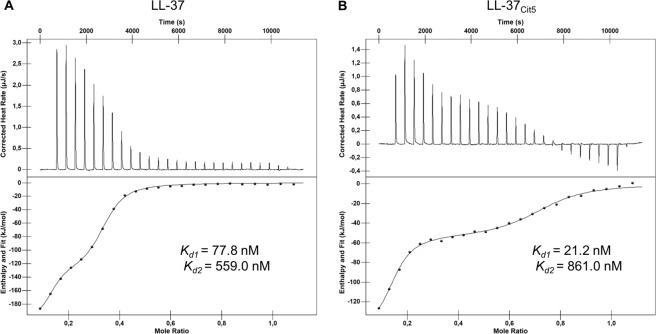
The interaction of native and citrullinated LL-37 with LPS. Analysis of ITC experiments with titrations of native LL-37 (**A**) or LL-37_Cit5_ (**B**) into LPS-O111:B4 solution. Every 300 seconds, 2 µL peptide in 0.1% TFA solution (200 µM) was titrated into 164 µL LPS solution (62.5 µM). Measurement of heat rate (*top panel*) and normalized integrated heat against molar ratio between LPS and the peptides (*lower panel*). Data of two independent experiments were fitted to calculate the dissociation constant (*K*_*d*_). The values of the fitting parameters are shown in Table 3.

As bacterial membrane integrity was clearly affected by native LL-37, but not by LL-37_Cit5_, we examined whether intrinsic differences in interaction between these peptides and LPS could explain this effect. To characterize the binding of LL-37 and LL-37_Cit5_ with LPS *in vitro*, ITC was performed in 0.1% TFA. Titration of LL-37 or LL-37_Cit5_ into a solution of LPS showed clear binding between LPS and both native LL-37 (Fig. 3A) and LL-37_Cit5_ (Fig. 3B). Further analysis of the thermograph showed that the binding consisted of multiple steps, with an initial binding event at relatively low peptide concentrations followed by a second binding event at higher peptide concentrations (bottom panel). Analysis of the binding curves showed that both peptides exhibited similar affinity in both the first and second binding event, however, the second binding event showed a higher stoichiometry for LL-37_Cit5_ (n = 0.5) compared to native LL-37 (n = 0.2). Analysis of the binding energies showed that binding of native LL-37 was more enthalpy-driven, compared to LL-37_Cit5_, suggesting an important role for the charged residues on native LL-37 to initiate this binding. Furthermore, the first binding event showed a higher enthalpy for both native LL-37 and LL-37_Cit5_ compared to the second binding event, suggesting the initial binding at low concentrations involved interaction with charged residues on the LPS micelle surface, while the second binding event most likely involved increased interaction with the hydrophobic inner lipids of the LPS (Table 3).

**Table 3:** Binding affinity, enthalpy changes, and entropy changes for the interaction between native or citrullinated LL-37 with LPS as determined by ITC.

Peptide	*First binding event*				*Second binding event*			
	K_d_ (nM)	∆H (kJ/mol)	−T∆S (J/mol × K)	n	K_d_ (nM)	∆H (kJ/mol)	−T∆S(J/mol × K)	n
**LL-37**	77.8	−237	194	0.126	559	−102	64.4	0.246
**LL-37**_**Cit5**_	21.2	−155	109	0.129	861	−53.9	17.9	0.558

Kd, dissociation constant; ∆H, change in enthalpy; ∆S, change in entropy; and n; stoichiometry

### Citrullinated LL-37 has reduced binding affinity for anionic phospholipids

**Figure 4:**
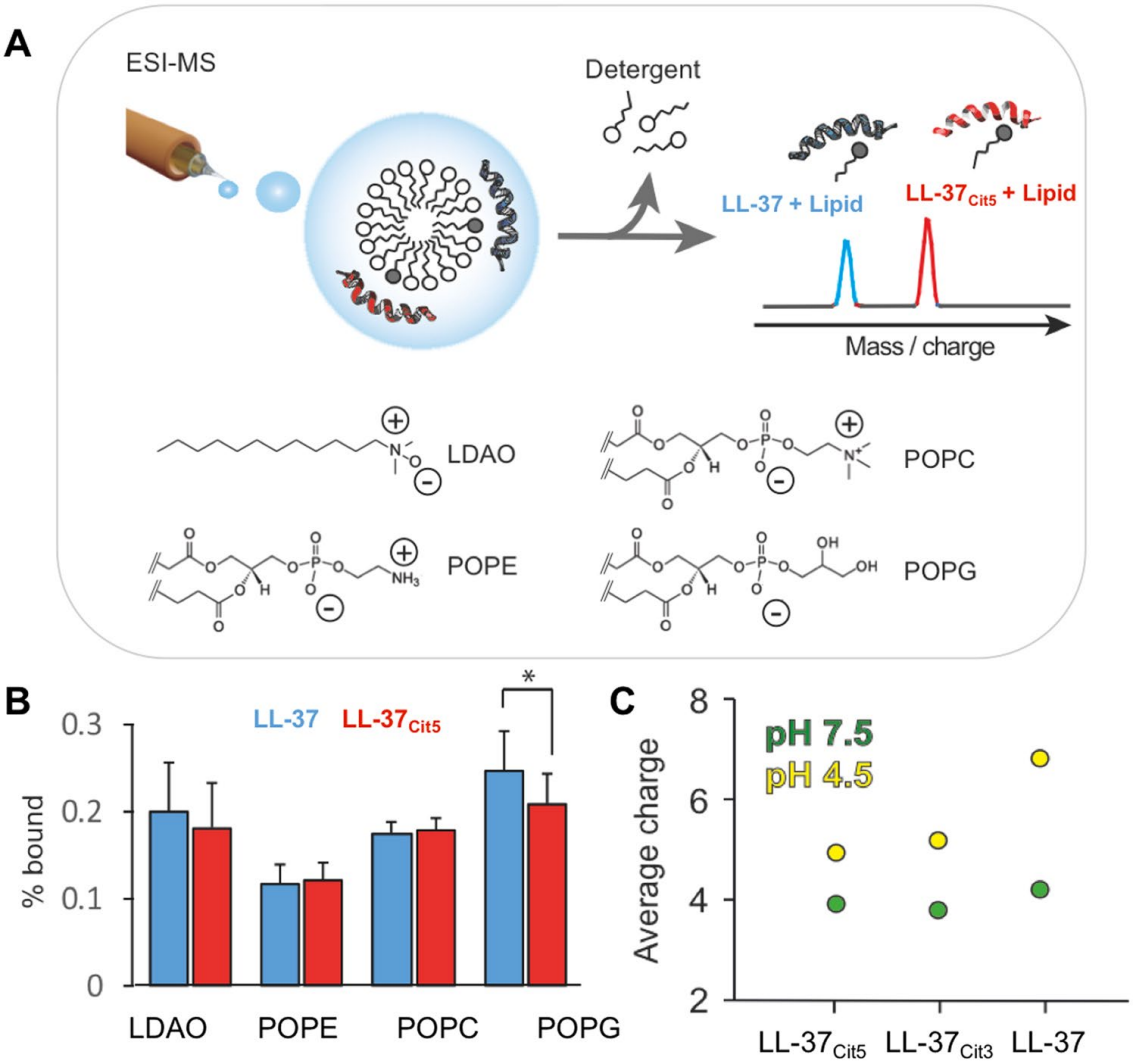
Analysis of lipid binding to native and citrullinated LL-37. (**A**) Schematic representation of the experiment design to study lipid binding to the peptides. Micelles composed of one of the phospholipids (POPC, POPE, POPG) and detergent LDAO were mixed with both native and citrullinated LL-37. **(B)** A comparison of the binding capacity between LL-37 and LL-37_Cit5_ released from micelles showing free peptides and peptides bound to phosopholipid. (**C**) The average charge of the peptides at physiological (*green circle*) and acidic (*yellow circle*) pH. Student’s t-test for paired samples with equal variance was performed (data representative of 3 independent experiments). *p-*values represented by stars; (<0.05*, <0.01**, <0.001***, <0.0001****).

Next, we assessed the effect of citrullination on the structure and membrane binding properties of LL-37. Using native mass spectrometry, we investigated whether citrullination would affect the ability of LL-37 to bind to common bacterial membrane lipids (Fig. 4). Briefly, solutions containing both LL-37 and LL-37_Cit5_ were mixed with zwitterionic micelles containing POPE, POPC, or POPG and subjected to MS analysis. Using gentle ionization conditions, peptide-lipid complexes formed in solution could be detected by MS, which enabled us to directly determine the ratio of lipid-bound and lipid-free peptides for LL-37 and LL-37_Cit5_ (Fig. 4A). We observed that both peptides readily bind to the zwitterionic detergent as well as to the zwitterionic lipids POPE and POPC to the same extent. However, the citrullinated peptide yielded fewer complexes with the negatively charged POPG than native LL-37 (Fig. 4B). To better understand the origin of the difference in lipid binding ability, we analyzed native LL-37 and two citrullinated forms (partially citrullinated LL-37_Cit3_ and fully citrullinated LL-37_Cit5_) under physiological (pH 7.5) and acidic (pH 4.5) conditions using mass-spectrometry. At physiological pH, all three peptides exhibited the same average charge. However, at acidic pH, the unmodified peptide acquired a significantly higher number of charges, while citrullinated forms retained the same low charge state (Fig. 4C). Together, our data indicate that reducing the net charge of the peptide through arginine citrullination interfered with its ability to interact with negatively charged phospholipid head-groups.

### Citrullinated LL-37 displays increased thermal stability compared to native LL-37

**Figure 5:**
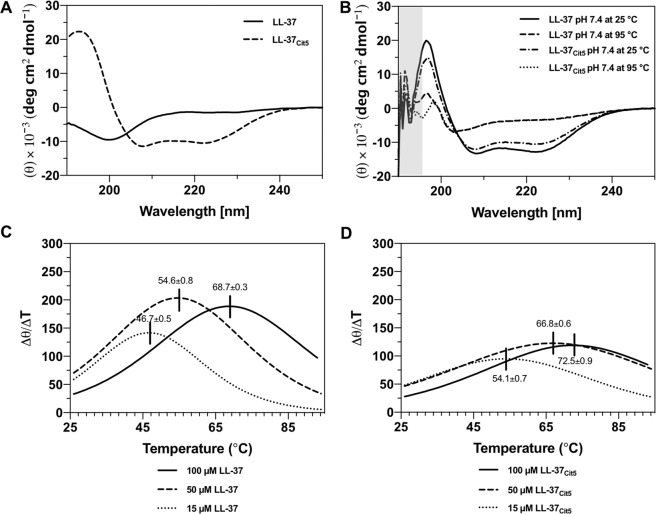
Analysis of conformational changes of the LL-37 and LL-37_Cit5_ peptides at different conditions by circular dichroism spectroscopy. **(A)** CD spectra were recorded for 50 µM LL-37 and 50 µM LL-37_Cit5_ peptides in water alone without buffer contributions. Under these conditions the native LL-37 displays a random coil structure, in contrast to the LL-37_Cit5_ peptide. When buffer (10 mM, pH 7.4) was added, native LL-37 forms α-helical secondary structures instantly. (**B**) CD spectra were recorded for 100 µM LL-37 and 100 µM LL-37_Cit5_ peptides in 10 mM PBS buffer (pH 7.4). The data points with a gray shaded area in (**B**) are not reliable due to high absorbance of the buffer. A smoothing function of 7 points was applied for the spectra. First derivative of temperature melting curves of native LL-37 (**C**) and LL-37_Cit5_ (**D**). Thermal melting profiles of the peptides were recorded at one single wavelength at 222 nm from 25–95 °C to follow the unfolding temperature dependence at pH 7.4 in PBS and sodium phosphate buffer. The maximum of the first derivative of the melting curve corresponds to the midpoint of a signal melting curve, and is interpreted as the melting temperature (T_m_).

To obtain further mechanistic insight into the folding, secondary structures and thermal stability of LL-37 and LL-37_Cit5_, CD spectrometry was used. The CD spectra recorded in Milli-Q water alone without any buffer at 25 °C for both peptides revealed a mainly random coil spectrum of LL-37 in agreement with previous findings^10,11^ and an α-helical spectrum for LL-37_Cit5_ with a single maximum at 195 nm and double minima at 222 and 208 nm, which is a characteristic of α-helical secondary structures (Fig. 5A)^22^. Upon addition of 10 mM PBS buffer pH 7.4 at 25 °C, both native LL-37 and LL-37_Cit5_ at three different concentrations (15, 50, and 100 μM) show α-helical secondary structures (Fig. 5B, & Table S2), in agreement with an earlier study^10^. At 95 °C in 10 mM buffer pH 7.4, both peptides unfolded into random-coil structures and were able to refold back to the original α-helical structures in buffer upon cooling to 25 °C (Fig. 5B). The melting temperature (T_m_) was estimated by recording the intensity at a single wavelength at 222 nm from 25 to 95 °C at pH 7.4. For 100 μM LL-37, the T_m_ was estimated to approximately 69 °C. Lower concentrations of LL-37 (15 μM and 50 μM) showed reduction in melting temperatures to 47 °C and 55 °C, respectively (Fig. 5C), reflecting a concentration dependent formation of oligomeric peptide aggregates^9^. The melting temperature of 100 μM LL-37_Cit5_ was generally higher than that of native LL-37, with 73 °C (Fig. 5D) and with lower concentrations of LL-37_Cit5_ (15 μM and 50 μM), the melting temperature decreased to 54 °C and 67 °C, respectively, again suggesting the presence of oligomeric peptide forms. Similar results of α-helical secondary structures and thermal stability was observed at low pH of 4.6 (Fig. S2). In general we observed that higher concentrations of peptides resulted in higher melting temperatures; furthermore citrullination increased the thermal stability of LL-37 at pH 7.4. Collectively, these results showed that the LL-37_Cit5_, with a loss of 5 positive charges, was slightly more temperature stable in its helical configuration, possibly as a result of increased hydrophobic interactions within and between the peptides.

### Negatively charged LUVs increased the helical content of citrullinated LL-37

**Figure 6:**
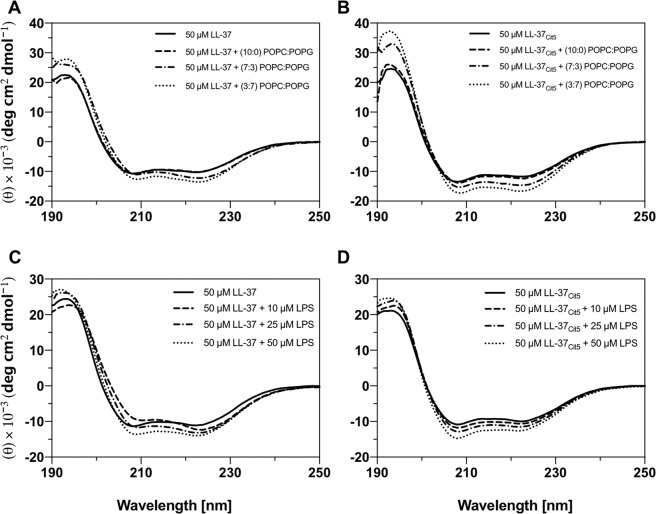
Analysis of conformational changes of LL-37 and LL-37_Cit5_ in LUVs and LPS by circular dichroism spectroscopy. CD spectra were recorded for 50 µM LL-37 **(A)** and 50 µM LL-37_Cit5_ **(B)** in 10 mM potassium phosphate buffer pH 7.4 in the absence or presence of 1 mM LUVs under quiescent conditions at room temperature. LUVs were composed of either POPC:POPG (10:0), POPC:POPG (7:3), or POPC:POPG (3:7). Titration of LPS onto 50 µM LL-37 **(C)** and 50 µM LL-37_Cit5_ **(D)** in 10 mM sodium phosphate buffer pH 7.4 at 25 °C recorded using CD spectroscopy. A smoothing function of 3 points was applied for all the spectra.

Large unilamellar vesicles (LUVs) with varying lipid compositions, mimicking a prokaryotic membrane, were prepared to investigate the impact of model membranes on the secondary structure of native and citrullinated LL-37 peptides. The secondary structures of LL-37 and LL-37_Cit5_ were determined by CD with and without the presence of LUVs. The α-helical content was determined by the signal intensity at 222 nm. The CD spectra of the peptides in the presence of zwitterionic POPC LUVs revealed a minor decrease in the α-helical content from 34% to 31% for LL-37 (Fig. 6A) and from 37% to 36% for LL-37_Cit5_ (Fig. 6B), but the θ_222_/θ_208_ ratio did not change. An increase in the negative charge of the liposomes to 30% (POPC/POPG, 7:3) did not change the α-helical content of native LL-37 (Fig. 6A), whereas for LL-37_Cit5_ the α-helical content slightly increased from 36% to 41% (Fig. 6B). However, the ratio between θ_222_/θ_208_ for native LL-37 increased for 30% liposomes. When the negative charge of the liposomes was increased to 70% (POPC/POPG, 3:7), the α-helical content of both peptides increased, from 36% to 38% for LL-37 (Fig. 6A) and from 40% to 45% for LL-37_Cit5_ (Fig. 6B). Again, the θ_222_/θ_208_ ratio for native LL-37 increased. Overall, we observed that the presence of negatively charged liposomes had minor impact of the α-helical content, although the helical content of citrullinated LL-37 increased to a higher extent than for the native peptide. The outer leaflet of Gram-negative bacteria is mainly composed of LPS, thus, we studied the secondary structure of both peptides in the presence of LPS molecules. Both peptides exhibited a slight increase in helicity through the increase of LPS concentration (Fig. 6C,D). However, titration of LPS onto LL-37 changed the intensity of mainly one of the typical alpha-helical minima (208 nm), whereas, titration of LPS onto LL-37_Cit5_ did not induced any substantial changes of the minimum at 208 nm. In general changes in the the θ_222_/θ_208_ ratio suggest a minor change in the α-helical structure, sometimes caused by so called helix supercoiling^23^. Hypothetically this could mean that the LL-37 peptide undergoes aggregation on the LPS surface, wheras LL-37_Cit5_ does not. After the final titration step both peptides were incubated in the presence of LPS over time. The LL-37_Cit5_ peptide was stable over the incubation time whereas the spectra of the native LL-37 peptide showed small increases of signal intensity at 208 nm (Fig. S3).

## Discussion

Here we could show that citrullinated LL-37 indeed is present in the human airways. This has been postulated in previous work, but not formally proven^15,24^. In order to detect citrullinated LL-37, we utilized cell-free BAL fluid obtained from from healthy human volunteers after intra-bronchial exposure to LPS^21,25^. We found that the BAL fluid from LPS-exposed lungs contained low, but clearly detectable, levels of citrullinated LL-37, which was demonstrated by using an in-house polyclonal serum raised against LL-37_Cit5_ combined with validation by a sensitive targeted LC-MS/MS analysis. Although the fragmentation pattern of LL-37 was rather complex, we were able to distinguish between two citrullinated forms of LL-37 (LL-37_Cit3_ and LL-37_Cit5_) in the BAL fluid fractions. It is important to note that neither dot blot analysis nor LC-MS/MS analyses can be considered as fully quantitative, but should rather be used to determine the presence or absence of the peptides in the samples. Therefore, our data provide the first proof-of concept-study where the *bona fide* citrullinated forms of LL-37 peptide have been detected directly in biological samples. It should be noted, however, that the polyclonal anti-serum raised against LL-37_Cit5_ exhibited cross-reactivity against LL-37_Cit3_ (data not shown). Thus it cannot be excluded that the initial screen revealed additional forms of citrullinated LL-37 that we could not distinguish with the LC-MS/MS analysis, due to analytical constraints. In fact, a previous paper showed that several proteolytic variants of LL-37 were present in the human epidermis^26^. Combined with the knowledge that citrullination increase proteolytic cleavage of LL-37, it is not unexpected that we also detected several variants of citrullinated LL-37^15^.

Native LL-37 exhibits antimicrobial activity against a wide range of Gram-negative and Gram-positive bacteria^12^. However, studies on citrullinated LL-37 are limited. A previous study showed that LL-37_Cit5_ exhibited weak antibacterial activity against the Gram-positive bacteria *S. pneumoniae* and *S. aureus*, and the Gram-negative ﻿non-typable *H. influenzae*. Furthermore, LL-37_Cit5_ displayed killing of *P. aeruginosa*, but the effect was slightly lower compared to native LL-37^15^. Here we found that citrullination of LL-37 at more than one arginine residue severely impaired the antibacterial activity against *E. coli* and prevented the permeabilization of the bacterial inner membrane, even in the presence of relatively high concentrations (Fig. 2). In addition, we found that fluorescently labelled LL-37_Cit5_ showed strong reduction in binding to *E. coli* compared to native LL-37, using confocal microscopy (Fig. 2). In this study, we also showed that LL-37_Cit5_ did not cause lysis of human erythrocytes, even up to 20 μM (Fig. S1). This suggests that the positive charges in the native peptide are crucial for the antibacterial activity. In general, the mechanism of cell-lytic activities of AMPs are mainly due to their ability to bind to the cell surface and to permeabilize the membrane leading to leakage of cell contents, resulting in cell death^11,27^. By a series of biochemical and biophysical experiments we attempted to dissect differences between native and citrullinated LL-37.

First, given that the initial interaction between LL-37 and the bacterial membrane is considered to be electrostatic, we investigated the interaction of citrullinated LL-37 to LPS, a major component of the Gram-negative bacterial outer membrane. Although citrullination decreased the net positive charge of the peptide, our ITC analysis indicated that the binding affinities to LPS were similar for both the native and citrullinated peptides. However, the slight shift to less enthalpy-driven binding as well as a higher stoichiometry for the interaction between LL-37_Cit5_ and LPS suggests that the binding of LL-37_Cit5_ to LPS is more hydrophobic in nature and altered compared to the interaction between native LL-37 and LPS. However, these results are not in line with the binding studies using dot blot and confocal on live intact bacteria, which was consistant with the killing of *E.coli*. The LPS-binding data from the ITC experiments could be explained by the fact that LPS micelles are different from actual bacterial membranes. Another explanation could be that regions of the LPS - possibly hydrophobic regions, such as lipid A - could be exposed to the peptides but not when it is attached to the bacterial membrane. Therefore, binding to LPS appears not to be enough for killing the bacterium, since the peptide needs to move towards the inner membrane in order to cause permeabilization. Thus, our data suggest that LL-37_Cit5_ might be unable to kill *E. coli* due to the loss of net charge, which in turn prevents the accumulation of the peptide on the bacterial outer membrane. However, it should be noted that there are other biophysical parameters that could affect peptide activity against bacteria, such as hydrophobicity, amphipathicity, oligomer formation and also the composition of the target membrane, which adds another layer of complexity to the peptide-membrane interactions.

Notably, other studies have shown that citrullination alters the immunomodulatory activity of LL-37, since its ability to neutralize LPS-induced proinflammatory cytokines by macrophages was reduced^15^. It was hypothesized that this reduced activity was because LL-37_Cit5_ binds poorly to LPS due to loss of its net charge, but formal proof of direct LPS binding has not been provided^15,19^. Therefore, it is possible that LL-37_Cit5_ can bind LPS mainly by virtue of hydrophobic interactions and still lack antibacterial activity due to the loss of net charge. It could be interesting in future studies, to investigate the binding location of LL-37_Cit5_ to LPS compared to native LL-37 to further understand the inhibition of *E. coli* killing by LL-37_Cit5_.

Next, we examined the interaction of LL-37_Cit5_ with bacterial phospholipids, another important component of bacterial membranes. Utilizing mass spectrometry, we found lower binding of LL-37_Cit5_ to the negatively charged phospholipids of the bacterial membrane as mimicked by POPG, when compared to native LL-37. CD analysis of LL-37_Cit5_ revealed that it adopted a typical α-helical structure at both physiological pH of 7.4 and at a lower pH of 4.6, suggesting that charge alone is not essential for α-helical secondary structures. Notably, native LL-37 requires a helical fold for antibacterial activity^10^, however, formation of a helix by LL-37_Cit5_ was not enough to retain antibacterial activity against *E. coli*.

The outer membrane leaflet of Gram-negative bacteria is mainly composed of LPS, wheareas cytoplasmic membranes are made of POPE and to a lesser extent by POPG which contribute to a negative net charge of the bacterial membrane^28^. Previous data has shown that LL-37 interacts with bacterial-mimicking membranes and adopts a helical secondary structure^9,11,15,29,30^. In line with these results, we found that LL-37_Cit5_ interacted with membrane models in a similar manner to the native form of the peptide. Combined, our results clearly show that LL-37_Cit5_ is deficient in activity against *E. coli*, which most likely is due to the reduced net charge. However, despite the reduced charge we still observed that LL-37_Cit5_ was able to bind LPS and bacterial mimicking phospholipids. This somewhat unexpected finding is consistent with an increased hydrophobicity of the citrullinated peptide.

In conclusion, we here identified citrullinated variants of LL-37 in the airways of healthy volunteers after intra-bronchial exposure to a LPS. In fact this is the first time that citrullinated LL-37 has been found in a human biological sample. Citrullination of LL-37 severely impaired its antibacterial activity against *E. coli*, most likely due to a reduced net charge. Nevertheless, the citrullinated peptide formed a typical α-helical structure and bound to LPS and bacterial-mimicking membranes in a manner similar to native LL-37, probably by virtue of hydrophobic interactions. What could be the role for citrullination in the context of the human airways? One aspect is to consider citrullination of histones, a key process in gene-transcription, but also essential for NETosis, which is an important host defense mechanism against bacteria^18^.

Previous reports have suggested that LL-37 may have a role in regulating inflammation in autoimmune diseases, such as psoriasis, systemic lupus erythematosus and arthritis^31^. Citrullination of LL-37 have been shown to increase the chemotactic activity towards leukocytes, whereas its ability to neutralize LPS-induced macrophage activation was suppressed. In addition, citrullination inhibited the anti-inflammatory effect of LL-37 on modulation of signaling via TLR2 and TLR3^19^. In addition, citrullination reduced the ability of LL-37 to form complexes with DNA, which in turn affected the ability to activate dendritic cells and macrophages by bacterial DNA^24^. Interestingly, autoantibodies against both native and citrullinated LL-37 have been detected in synovial fluid and plasma of psoriatic arthritis patients, which suggests that LL-37 play a role as autoantigens in this disease^32^. A recent report showed that circulating LL-37 is associated with anti-cyclic citrullinated peptides (anti-CCP) antibodies in early inflammatory arthritis, suggesting that LL-37 may be involved in the development of the disease^33^. Thus, it is possible that citrullination of LL-37 represents a collateral and unwanted effect that dramatically alter the antimicrobial effect of this AMP. In addition, citrullination alters the immunomodulatory functions of LL-37 and it is possible that citrullinated LL-37 can serve as a neoantigen, thus breaking tolerance and driving the autoimmune process. From the positive side it could instead be argued that citrullination represents a key step to “control” an overreactive AMP-based immune-system; a hypothesis that is supported by data showing that citrullination turns LL-37 into a better substrate for proteolytic enzymes^15^. Altogether, our results can pave the way to study the role of citrullinated LL-37 in infectious and inflammatory diseases, such as pneumonia, sepsis, chronic obstructive pulmonary disease, psoriasis, and cystic fibrosis, but also in normal physiology.

## Materials and Methods

### Reagents

Blood agar plates, phosphate buffered saline (PBS), and Luria Bertani (LB), were obtained from the substrate unit at the Karolinska University Hospital, Stockholm, Sweden. Lyophilized synthetic LL-37, citrullinated LL-37 peptides (Table 1), 5-FAM-LL-37 and Rhodamine B-LL-37_Cit5_ peptides were purchased from Innovagen (Lund, Sweden) and dissolved in 0.1% triflouroacetic acid (TFA), aliquoted and stored at −20 °C until use.

### Intra-bronchial LPS exposure and collection of bronchoalveolar lavage fluid

The cell-free BAL fluid samples were obtained as part of a clinical study performed from 11^th^ of November 2003 to 3^rd^ of December 2008^21,25^. This sample collection constituted a part of a larger project studying the innate immune response in healthy human airways with and without prior intra-bronchial LPS exposure to LPS, from which other results have already been presented^21,25^. This clinical study was performed in accordance with the Helsinki declaration after approval by the ethics committee Regional Ethical Review Board in Gothenburg, Sweden (Dnr. 618-02, 065-04 and 683-07). Written informed consent was obtained prior to inclusion in the study. In brief, healthy volunteers were exposed via bronchoscopy to vehicle, *i.e*., 10 mL of 0.9% PBS in one lung and to LPS (4 ng/kg) from *Escherichia coli* (*E. coli*) 0113:H10 (Rockville, MD, USA), diluted in PBS, in the other lung. After either 12 or 24 h after the exposure to vehicle and LPS, respectively, BAL was performed (3 × 50 mL of PBS at 37 °C) in the respective lung. After additional processing as described elsewhere^21,25^, the cell-free BAL fluid samples were stored at −80 °C until further use. For the current study, we utilized BAL samples from LPS-exposed lungs only.

### Peptide/protein extraction from BAL fluid samples

The BAL fluid samples from LPS-exposed airways were pooled together (material from n = 10 healthy volunteers, total of 14 mL), cleared from debris by centrifugation (5,000 rpm for 15 min) and reconstituted in 0.1% TFA. The supernatants were enriched for peptides and proteins utilizing an OASIS 1cc reversed-phase HLB column (Waters Corp., Milford, MA, USA). The OASIS columns were activated by acetonitrile and equilibrated in aqueous 0.1% TFA before the supernatants were applied. Unbound material was washed away with 0.1% TFA and bound material was eluted with 80% acetonitrile in 0.1% TFA. The eluated fractions were frozen at −80 °C, lyophilized overnight and stored at −20 °C until further use.

### Reversed-phase liquid chromatography

The peptide/protein extracts (0.5 mg) were separated on a Vydac C_8_ (250 × 4.5 mm) reversed-phase column (HiChrom Ltd, UK) with a flowrate of 0.3 mL/min. Elution was performed with an increasing gradient of acetonitrile in 0.1% TFA (0–20% for 16.6 min, 20–60% for 33.2 min and 60–80% for 66.2 min). The absorbance was monitored at 214 nm and 280 nm. Sixty two fractions of 1 mL were collected, immediately frozen (−80 °C) and lyophilized.

### LC-MS/MS analysis

The synthetic peptides (LL-37 and LL-37_Cit5_) were dissolved in 2% acetonitrile/0.1% formic acid (solvent A) and injected separately on an Easy nano LC-II HPLC system (Thermo Scientific, Bremen, Germany) on-line coupled to a Q Exactive mass spectrometer (Thermo Scientific, Bremen, Germany). The synthetic peptides of native and citrullinated LL-37 were used as a reference to determine their ionization and fragmentation properties. The chromatographic separation of the peptides was achieved using an analytical column packed with ReproSil-Pur C8, 3 µm particles (Dr. Maisch, Ammerbuch, Germany) in 10 cm of a PicoTip silica emitter with 75 µm inner diameter (New Objective, Woburn, MA, USA). To separate the peptides, a gradient of increasing acetonitrile concentrations (4–25% for 5 min, 25–70% for 5 min, 70–95% for 5 min and at 95% for 8 min) was applied at a flow rate of 300 nL/min. The MS acquisition method was comprised of tandem mass scans in parallel reaction monitoring (PRM) mode in a mass range *m/z* 300 to 1650, while targeting three precursor *m/z* values defined in the inclusion mass list (LL-37: *m/z* 749.4365 [M + 6 H]^6+^; LL-37_Cit3_: *m/z* 899.7128 [M + 5 H]^5+^; LL-37_Cit5_: *m/z* 900.1064 [M + 5 H]^5+^). The mass resolution was set to 17.500 (at *m/z* 200) for the high collision dissociation (HCD) at 35% normalized collision energy, fragmenting the 15 most intense precursor ions isolated with *m/z* 1.6 width, targeting 10^5^ ions. The lyophilized BAL fluid fractions were dissolved in 20 µL of solvent A and 2 µL was injected on the column. The acquired data was analyzed in Skyline v.4.2 (MacCoss Lab, University of Washington).

### Dot blot analysis of chromatographic fractions

For dot blot analysis, the lyophilized chromatographic fractions were dissolved in 30 μL 0.1% TFA and 1 μL from each fraction was dotted on Hybond Super C membrane (Amersham, GE Healthcare UK Ltd., Little Chalfont, UK). The membranes were blocked in 5% fat-free milk in PBS with 0.25% Tween-20 and then incubated overnight at 4 °C with either primary mouse monoclonal antibody against LL-37 (1.2 mg/mL; 1: 2000 dilution in PBS with 5% fat-free milk)^34^ or with an affinity purified polyclonal serum raised against LL-37_Cit5_ (5.4 mg/mL 1: 2000 dilution in PBS with 5% fat-free milk) from Innovagen (Lund, Sweden). Subsequently, after a washing step, membranes were incubated with secondary antibodies coupled to horseradish peroxidase (Sigma-Aldrich, St. Louis, MO, USA) corresponding to each primary antibody (1 h, RT). The signal was developed by the ECL prime system (Amersham, GE Healthcare UK Ltd.) and detected by a Syngene digital imaging system (Syngene, Cambridge, UK). Immunoblot images were adjusted using GeneSys software (Syngene, Cambridge, UK).

### Bacterial culture

*Escherichia coli* ATCC 29522 (*E. coli*) was obtained from the clinical microbiology laboratory at Karolinska University Hospital. Bacteria were grown in Luria Bertani (LB) broth at 37 °C with shaking (200 rpm) to reach mid-logarithmic phase, after which the bacterial suspension were diluted to the starting bacterial inoculum in the same growth medium prior to each experiment.

### Colony count assay

The antibacterial activity of LL-37 and the citrullinated LL-37 peptides (Table 1) was assessed by performing a colony counting assay. *E. coli* was grown to mid-logarithmic phase in LB, and subsequently diluted to 5 × 10^7^ CFU/mL. Bacteria were incubated with different peptide concentrations (0 to 80 μM) for 3 h at 37 °C. Subsequently, mixtures were serially diluted and spot-plated (20 μL) on blood agar plates. Surviving bacteria were counted after an overnight incubation at 37 °C.

### Sytox green uptake assay

To assess inner membrane permeabilization, 5 × 10^7^ CFU/mL live or heat-killed *E. coli* was incubated with LL-37 or LL-37_Cit5_ for 30 min at 37 °C in LB. After incubation, bacteria were centrifuged at 1,200 × g for 10 min at 4 °C and washed in PBS. Next, bacteria were resuspended in PBS with 3 μM SYTOX Green Nucleic Acid Stain (Thermo Fisher Scientific,Waltham, MA, USA) and transferred to a black 96-well plate. After 5 min incubation at room temperature, a TECAN microplate reader was utilized to measure the fluorescence intensity at two wavelengths (λ_ex_ 504 nm and λ_em_ 523 nm).

### Transmission electron microscopy

*E. coli* was grown to log phase and diluted to 5×10^8^ CFU/mL in LB. To adjust for the higher bacterial concentrations that were required for electron microscopy, additional colony count assays were performed to define the antibacterial capacity of LL-37 at this bacterial concentration. The minimum inhibitory concentration of LL-37 was 50 μM. In contrast, LL-37_Cit5_ did not exhibit any killing even at higher concentrations (200 μM). Untreated bacteria were used as controls and incubated with either LL-37 or LL-37_Cit5_ at different concentrations (50 μM or 200 μM) at 37 °C for 0.5 or 2 h. Mixtures were fixed in 2.5% glutaraldehyde in 0.1 M phosphate buffer, pH 7.4 at room temperature for 30 min and then rinsed in 0.1 M phosphate buffer prior to post-fixation using 2% osmium tetroxide in 0.1 M phosphate buffer, pH 7.4 at 4 °C for 2 h. Next, the samples were dehydrated in ethanol followed by acetone and finally embedded in LX-112. Ultrathin sections were prepared using a Leica EM UC7 (Leica Microsystems GmbH, Wetzlar, Germany) and uranyl acetate was used as a contrast followed by Reynolds lead citrate. The sections were examined in a Tecnai Spirit G2 Bio TWIN Electron microscope (Tecnai High-Technologies) at 100 kV and images were acquired using a 2kx2k Veleta CCD camera (Olympus Soft Imaging Solutions GmbH, Münster, Germany).

### Confocal microscopy

For confocal microscopy, 10^7^ CFU/ml *E. coli* were incubated with labelled peptides 5-FAM-LL-37 or Rhodamine B-LL-37_Cit5_ (Innovagen) for 1 hour at 37 °C in LB. After incubation, bacteria were centrifuged at 5,000 × *g* for 2 min and washed in PBS twice. Next, bacteria were fixed in 2% PFA (Sigma-Aldrich) for 30 min at room temperature. Bacteria were washed and resuspended in PBS, and pipetted onto a microscopy slide, air-dried, heat-fixed, and mounted in ProLong Gold Antifade mountant (Thermo Fisher Scientific). Confocal imaging was performed on an Olympus FluoView FV1000 confocal laser scanning microscope using Olympus FluoView software to acquire images. Image analyses were performed using ImageJ/FIJI software (National Institutes of Health, Bethesda, MD, USA).

### Hemolysis assay

Human blood was collected in heparin-sodium blood collection tubes (Becton Dickinson, Plymouth, UK). Whole blood was centrifuged at 800 × *g* for 10 min at room temperature. Plasma was discarded and erythrocytes were washed three times with PBS (pH 7.4), then resuspended to 1% (vol./vol.) solution in PBS. Erythrocytes were analyzed in a U-bottom plate, mixed with different peptide concentrations (1.25–20 µM) and incubated for 1 hour with agitiation at 37 °C. The plate was centrifuged at 800 × *g* for 10 min and 80 µL supernatant was transferred to a clear flat bottom 96-well plate to measure absorbance of hemoglobin released at 540 nm using a Tecan Infinite M200 microplate reader. The percent of hemolysis was calculated using a negative (no peptides) as 0% lysis and a positive control (1% Triton X-100) as 100% lysis.

### Isothermal titration calorimetry

Isothermal titration calorimetry (ITC) was performed with the Low Volume NanoITC (TA Instruments-Waters LLC, New Castle, DE, USA). The 50 µL syringe was filled with 200 µM LL-37 or 200 µM LL-37_Cit5_ in 0.1% TFA (Sigma-Aldrich) for titration into 164 µL of 62.5 µM *E. coli* O111:B4 LPS (InVivoGen, San Diego, CA, USA) in 0.1% TFA (Sigma-Aldrich). Titrations were incremental with 2 µL injections at 300 s intervals. Experiments were performed at 37 °C. Data were analyzed with the NanoAnalyze software (TA Instruments-Waters LLC, New Castle, USA).

### Mass spectrometry

Prior to MS analysis, both native and citrullinated peptides were diluted ten times in 1 M ammonium acetate, pH 7.5, or 0.1% acetic acid, pH 4.5. For lipid binding experiments, the peptides were diluted in 1 M ammonium acetate, pH 7.5, containing 4 mM of detergent lauryldimethylamine N-oxide (LDAO) to a final concentration of 5 µM each. In brief, zwitterionic (neutral charge) 1-palmitoyl-2-oleoyl-sn-glycero-3-phospho-choline (POPC), 1-palmitoyl-2-oleoyl-sn-glycero-3-phosphoethanolamine (POPE) and negatively charged 1-palmitoyl-2-oleoyl-sn-glycero-3[phospho-rac-(1-glycerol)] (POPG) were obtained from Avanti Polar Lipids (Alabaster, Alabama, USA) with a concentration of 800 µM each were prepared in Milli-Q water. For binding studies, each phospholipid was added to the mixture of peptides in LDAO to a final concentration of 80 µM. Mass spectra were recorded on an Orbitrap Fusion (Thermo Fisher Scientific, Waltham, MA) equipped with an offline nano-electrospray source. Samples were introduced using gold-coated Proxeon borosilicate capillaries (Thermo Scientific, Waltham, MA). Spectra were recorded in a positive ionization mode with a capillary voltage of 1.8 kV and a source temperature of 80 °C. Data were analyzed using the Xcalibur software 3.0 package (Thermo Scientific). Student’s T-test for paired samples with equal variance was performed in Microsoft Excel.

### Circular dichroism (CD)

#### Sample preparation

Synthetic lyophilized LL-37 and LL-37_Cit5_ peptides were dissolved in Milli-Q water, the peptide concentration was determined by the weight of the dry powder. Further dilutions were prepared in 10–50 mM PBS, citric acid buffer, sodium phosphate or potassium phosphate buffer to obtain the final peptide concentration used for the experiments.

#### Liposome preparations

To mimic the function of phospholipids in a physiological membrane large unilamellar phospholipid vesicles (LUVs) were used. The LUVs were prepared according to a previously published protocol^35^. In brief, neutral POPC and negatively charged POPG were mixed to a final phospholipid-concentration of 5 mM (for 30% negatively charged LUVs a POPC/POPG molar ratio of 7:3 was used, for 70% negatively charged LUVs POPC/POPG 3:7 was used, and for 100% neutral LUVs POPC 10:0 was used). Chloroform was evaporated under a stream of nitrogen for at least one hour. The lipid film was thereafter resuspended in 50 mM potassium phosphate buffer (pH 7.4) and vortexed for 10 minutes to form multilamellar vesicles. To obtain unilamellar vesicles the lipid solution was frozen in liquid nitrogen and thawed in hot water with a freeze/thaw cycle of five times, and thereafter passed through an Avanti mini extruder (Avanti Polar Lipids, Alabaster, Alabama, USA) with a 100 nm polycarbonate pore size filter (Avanti Polar Lipids, Alabaster, Alabama, USA) for at least 21 times. The LUVs solution was further diluted in potassium phosphate buffer pH 7.4 to obtain a final concentration of 1 mM.

#### LPS micelles preparation

Lyophilized LPS from *E. coli* O111:B4 was obtained from Sigma Aldrich. A 200 µM stock solution of LPS micelles was prepared by dissolving the lyophilized LPS in Milli-Q water. The stock solution was vortexed for one and a half minute. The LPS concentration was determined by weight, using a LPS molecular weight of 20 kDa.

#### Circular dichroism (CD) spectroscopy

CD spectra and temperature scans were recorded on a Chirascan CD spectrometer (Applied Photophysics, Leatherhead, U.K.) with a Peltier temperature control system. A quartz cuvette with a 1 mm pathlength was used. The secondary structures of the peptides were studied by recording CD spectra under quiescent conditions in the spectral range of 190–260 nm with a bandwidth of 1 nm, a step size/resolution of 0.5 or 1 nm, and a time-per-point of 0.5 or 4 s. Background spectra of the buffer were subtracted and the data were presented as mean residue molar ellipticity. All data obtained with a time-per-point of 0.5 s were processed with a smoothing function of three or seven points. The concentration of the peptides (15–100 µM), pH (4.6 and 7.4) and the temperature (5–95 °C) were varied and the changes in peptide secondary structures were followed. For the membrane mimicking experiments 1 mM LUVs in 10 mM potassium phosphate buffer pH 7.4 at room temperature was used. Titration steps of LPS (10–50 µM) into a 50 µM peptide solution were performed in 10 mM sodium phosphate buffer pH 7.35 at 25 °C. After the final titration step of 50 µM LPS the sample with peptides and LPS was allowed to incubate for 70 minutes at 25 °C while spectra were recorded every tenth minute. The spectra are presented after subtraction of a background spectrum of each LPS concentration in buffer. To calculate the α-helical content (Eq. 1) was used^36^

1 $${\rm{\alpha }}-{\rm{helical}}\,{\rm{content}}\,[ \% ]=(\frac{{{\rm{\theta }}}_{222{\rm{nm}},{\rm{random}}{\rm{coil}}}-{{\rm{\theta }}}_{222{\rm{nm}},{\rm{observed}}}}{{{\rm{\theta }}}_{222{\rm{nm}},{\rm{random}}{\rm{coil}}}-{{\rm{\theta }}}_{222{\rm{nm}},{\rm{\alpha }}-{\rm{helix}}}})\ast 100,$$

where the average ellipticity for random coil structures (θ_222 nm,random coil_) is 3.900 deg cm^2^ dmol^−1^ and the average ellipticity for α-helices (θ_222 nm,α-helix_) is −35.700 deg cm^2^ dmol^−1^.

The unfolding of the LL-37 and LL-37_Cit5_ peptides at different peptide concentrations and pH were followed by changes of the ellipticity at 222 nm as a function of temperature, in the temperature range of 25–95 °C. One single wavelength, 222 nm, was chosen due to the typical α-helical minimum at 222 nm. The ellipticity was measured with a step size of 1 °C with a rate of 5 °C min^−1^ or 0.3 °C min^−1^. The ellipticity changes at 222 nm can be used to calculate the thermodynamical parameters of unfolding. The melting temperature, T_m_, for each condition was determined by sigmoidal curve fitting of the data points (Eq. 2). The T_m_ values were calculated from the first derivative of the curves, where the maximum corresponds to the temperature scan midpoint (T_m_).

2 $$F(T)=A+\frac{B-A}{1+\exp [\frac{{T}_{m}-T}{w}]}$$

where A is the amplitude of the melting curve, B is the baseline, T_m_ is the midpoint or melting temperature and w is the slope of the curve.

### Statistical analysis

The CFU count data were log-transformed and subjected to an unpaired multiple T-test. Data represented as mean ± SEM. A *p*-value of ≤0.05 was considered as statistically significant. Statistical analysis, GraphPad Prism version 7.0e (GraphPad, La Jolla, CA) was used.

## Supplementary information

Supplementary Method.
