# Supplementary material for: Studies on citrullinated LL-37: detection in human airways, antibacterial effects and biophysical properties
Source: Sci Rep. 2020 Feb 11;10:2376. doi: 10.1038/s41598-020-59071-7 (PMC7012854; doi:10.1038/s41598-020-59071-7)
Supplement: Supplementary file 1 — Supplementary Method. [file 41598_2020_59071_MOESM1_ESM.docx]

**Supporting Information**

**Studies on citrullinated LL-37: detection in human airways, antibacterial effects and biophysical properties**

Salma Al Adwani, Cecilia Wallin, Melanie D. Balhuizen, Edwin J. A. Veldhuizen, Maarten Coorens, Michael Landreh, Ákos Végvári, Margareta E Smith, Ingemar Qvarfordt, Anders Lindén, Astrid Gräslund, Birgitta Agerberth, Peter Bergman

List of the materials included:

1. Figure S1
2. Figure S2
3. Figure S3
4. Table S1
5. Table S2

******

**Figure S1. Analysis of hemolytic activity of citrullinated LL-37.**

The hemolytic activity of native LL-37 (blue) and LL-37_Cit5_ (red) was analyzed on human red blood cells. Erythrocytes were incubated for 1 h with each peptide at concentrations from 1.25 to 20 µM. The hemolytic activity of the peptides was evaluated by measuring the release of hemoglobin at 540 nm. Triton (1% v/v) was set to 100% lysis and no peptide as a negative control (data representative of 3 independent experiments). Error bars show SEM. Statistical significance was evaluated by Mann-Whitney U-test: *p-*values represented by stars; (<0.05 *, <0.01 **, <0.001 ***, < 0.0001 ****).

S- 2


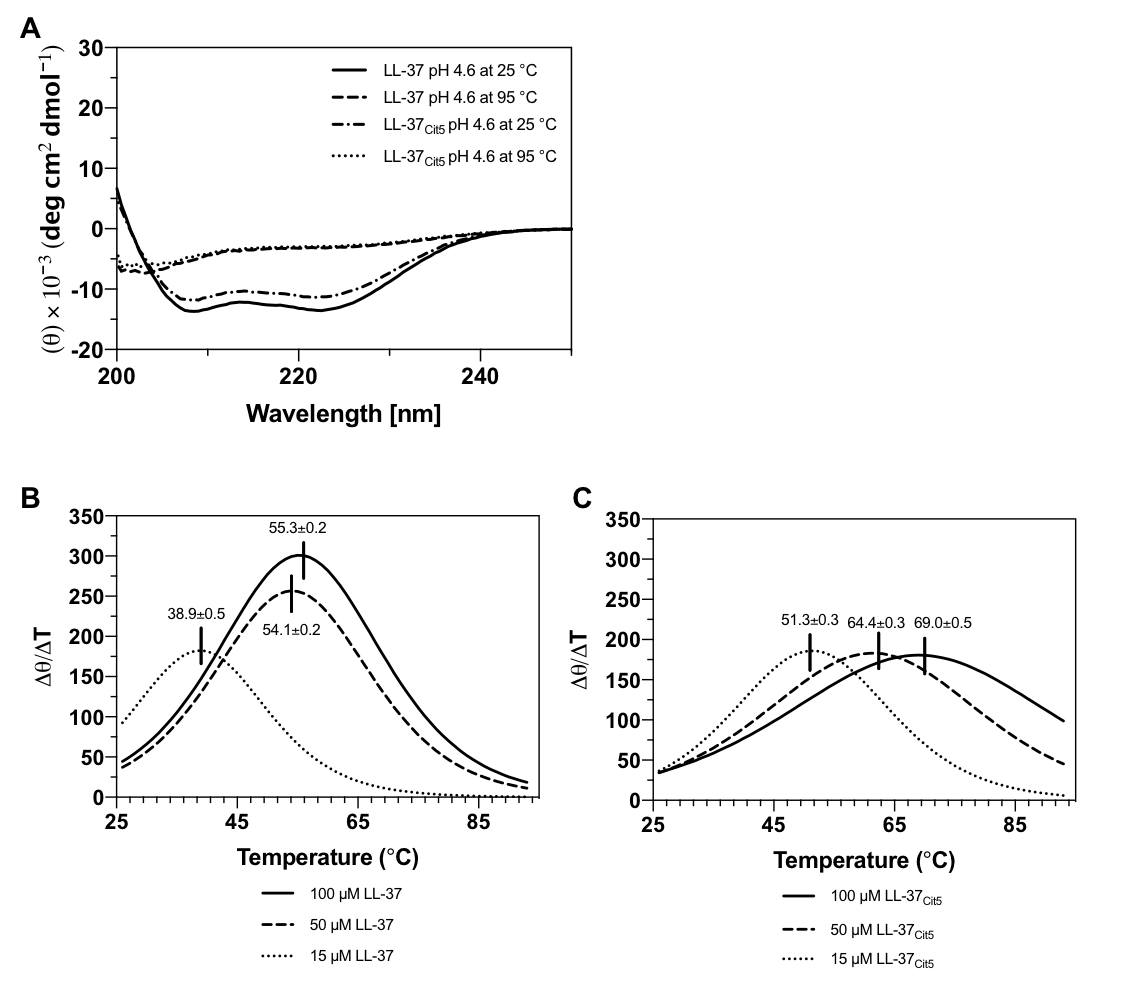


**Figure S2. Analysis of conformational changes of the LL-37 and LL-37_Cit5_ peptides at acidic conditions by circular dichroism (CD) spectroscopy.**

(*A*) CD spectra were recorded for 100 µM LL-37 and LL-37_Cit5_ peptides in citric acid buffer pH 4.6. A smoothing function of 7 recorded points was applied for the spectra. (*B, C*) First derivative of temperature melting curves. Thermal melting profiles were recorded at one single wavelength at 222 nm from 25-95 °C to follow the unfolding temperature dependence at pH 4.6. The maximum of the first derivative of the melting curve corresponds to the midpoint of a signal melting curve, and is interpreted as the melting temperature (T_m_).

LL-37

LL-37_Cit5_

**Figure S3. Analysis of conformational changes of the** **LL-37 and LL-37_Cit5_ peptides with LPS over time by circular dichroism spectroscopy.**

Samples with LL-37 and LL-37_Cit5_ peptides and 50 µM LPS after the final titration step in Fig. 6 C,D in the main manuscript were incubated for 70 minutes at 25 °C ,while spectra were recorded every tenth minute. The background of 50 µM LPS in buffer was subtracted from the spectra.

**Table S1.** Measured intensities of fragment ions of native and citrullinated LL-37 peptides found in BAL fractions analyzed by ESI-MS-PRM method.

|  |  | **Peak Area^*^** |  |
| --- | --- | --- | --- |
| **Fraction number** | **LL-37:** 749.4365, +6 ^**^ | **LL-37_Cit3_:** 899.7128, +5 ^**^ | **LL-37_Cit5_:** 900.1064, +5 ^**^ |
| F22 | 6.33×10^5^ | 4.9×10^5^ | 3.97×10^5^ |
| F23 | 1.23×10^5^ | 7.99×10^4^ | 1.53×10^5^ |
| F24 | 1.17×10^6^ | 2.19×10^6^ | 2.63×10^6^ |
| F25 | 0 | 6.27×10^2^ | 0 |
| F26 | 6.34×10^6^ | 3.32×10^6^ | 3.25×10^6^ |
| F27 | 2.06×10^8^ | 8.34×10^7^ | 6.04×10^7^ |
| F28 | 1.43×10^8^ | 5.82×10^7^ | 4.57×10^6^ |
| F29 | 2.13×10^7^ | 1.06×10^7^ | 8.71×10^6^ |
| F30 | 1.33×10^6^ | 8.66×10^5^ | 6.62×10^5^ |
| F31 | 0 | 0 | 1.55×10^3^ |

^*^ Combined peak areas of all fragment ions for each peptide.

^**^precursor ions of each peptide and the charge state detected by mass spectrometry.

**Table S2*.*** Prediction of the α-helical content of LL-37 and LL-37_Cit5_ peptides at different concentrations and temperatures determined from the ellipticity in the CD spectra.

|  | **pH 7.4** | |  | |  | **pH 4.6** | |
| --- | --- | --- | --- | --- | --- | --- | --- |
|  | **15 μM**  25C  95C  25C* | **50 μM**  25C  95C  25C* | | **100 μM**  25C  95C  25C* | **15 μM**  25C  95C  25C* | **50 μM**  25C  95C  25C* | **100 μM**  25C  95C  25C* |
| **LL-37**  **α-helical content [%]** | 32.6  19.8  34.2 | 41.1  18.5  40.2 | | 42.2  18.5  41.6 | 25.4 - - | 35.1 16.3 34.6 | 44.0 17.9 44.5 |
| **LL-37_Cit5_**  **α-helical content [%]** | 26.9  16.0  27.4 | 35.8  18.2  36.4 | | 36.4  18.6  34.7 | 30.7 15.8 32.0 | 36.3 16.8 37.4 | 38.5 17.3 39.5 |

^*^After refolding
